# Uip4p modulates nuclear pore complex function in *Saccharomyces cerevisiae*

**DOI:** 10.1080/19491034.2022.2034286

**Published:** 2022-02-16

**Authors:** Pallavi Deolal, Imlitoshi Jamir, Krishnaveni Mishra

**Affiliations:** a Department of Biochemistry, School of Life Sciences, University of Hyderabad, Hyderabad, India; b Department of Biotechnology, School of Engineering and Technology, Nagaland University, Dimapur, India

**Keywords:** Nuclear envelope, nuclear pore complex, yeast, Uip4

## Abstract

A double membrane bilayer perforated by nuclear pore complexes (NPCs) governs the shape of the nucleus, the prominent distinguishing organelle of a eukaryotic cell. Despite the absence of lamins in yeasts, the nuclear morphology is stably maintained and shape changes occur in a regulated fashion. In a quest to identify factors that contribute to regulation of nuclear shape and function in *Saccharomyces cerevisiae*, we used a fluorescence imaging based approach. Here we report the identification of a novel protein, Uip4p, that is required for regulation of nuclear morphology. Loss of Uip4 compromises NPC function and loss of nuclear envelope (NE) integrity. Our localization studies show that Uip4 localizes to the NE and endoplasmic reticulum (ER) network. Furthermore, we demonstrate that the localization and expression of Uip4 is regulated during growth, which is crucial for NPC distribution.

## Introduction

Eukaryotes have intracellular compartmentalization, and the biological processes are regulated spatially and temporally within membrane bound organelles. Nucleus is one of the most prominent organelles and an important defining feature of any eukaryotic cell. It harbors the genetic material within a double membrane bilayer that compartmentalizes it from the rest of the cellular components. The inner nuclear membrane (INM) acts as a site of regulation for various nuclear processes including transcription, mRNA export, ribosome biogenesis, and DNA repair. Several INM associated proteins also provide structural support to the nucleus [1,2]. The outer membrane (ONM) is continuous with the endoplasmic reticulum, which extends up to the cell periphery in yeast. The selective exchange of macromolecules between the nucleus and cytoplasm occurs through the macromolecular assemblies called the nuclear pore complexes (NPCs). NPCs are embedded in the NE where the inner and outer nuclear membrane fuse. The estimated mass of an NPC in yeast is ~60MDa comprising around 30 different types of nucleoporins [3] with most of these present as 16 copies per NPC [4,5].

-The NPC scaffold consists of three stacked rings, an inner ring that is sandwiched by two outer rings (cytoplasmic and nucleoplasmic), each of which has an eight-fold rotational symmetry [6]. The Nups are assigned to various classes primarily depending on their relative position in the entire complex [5–7]. The scaffold Nups (Yeast Pom152p, Pom34p, and Ndc1) are the ones that nucleate the NPC assembly and hold the entire complex in place resulting in a cylindrical core [8,9]. The phenylalanine-glycine (FG) repeat containing Nups are present along the inner side of the channel and are key regulators of the direction and flux of transport. The nucleoplasmic and cytoplasmic rings are concentric rings made of the Nup107-160 sub-complex (yeast Nup84 sub-complex). This comprises of Nup133p, Nup120p, Nup145Cp, Nup85p, Nup84p, Seh1p, and Sec13p. Several nuclear and non-nuclear components are important in regulating the assembly, function, and turnover of NPCs [10]. The ONM is known to share several proteins with the endoplasmic reticulum (ER). Some of these ER proteins, such as the reticulons- Rtn1 and Yop1, and membrane metabolism-related proteins, Brr6, Brl1, and Apq12, also contribute to NPC biogenesis and assembly [11–14].

NPC assembly is known to operate via two major pathways [15,16]. One is the *de novo* assembly during interphase when nucleoporins are recruited to a pore site at the nuclear membrane. Second, post-mitosis, when sub-complexes and membrane vesicles are recruited to NE during nuclear envelope reformation. In organisms that undergo closed mitosis, only the former mechanism takes place. Defective assembly of NPC intermediates and dysregulation of nuclear quality control pathway results in abnormal nuclear shape [17–19]. Mis-localization and altered stoichiometry of nucleoporins has been also linked to aging and clinical pathologies [20]. NE extension or herniations along with NPC clustering have been observed in yeast as a result of failed nuclear protein quality control pathways [21, Webster et al., Thaller et al., 22,23]. In many instances where NPC aggregation is seen, accompanying nuclear shape changes are also observed [21].

Presence of nups outside of the NE is atypical, but cytosolic spots containing NPC components have been reported under various physiological and non-physiological conditions in budding yeast [19,24–26]. While some of the NPC components might assemble in the cytosol, they are not recruited at the NE until the transmembrane and scaffold nups have created pore site for insertion. In yeast, some of these cytoplasmic spots are pools of nups and remain associated with lipid droplets [27]. While in other cases, these components are retained to prevent incorporation of damaged nups at the NE thereby protecting NE integrity [19]. Cytosolic spots of nups have been observed in yeast mutants that affect the assembly and stability of NPCs [8,28].

To identify novel components involved in maintaining nuclear architecture, we initiated a fluorescence microscopy-based genome-wide screen approach in budding yeast. Yeast serves as a good model for such a screen because unlike metazoans it undergoes closed mitosis and the yeast nuclear membrane does not disassemble during mitosis [29]. We found that the loss of certain physical interactors of Ulp1, a deSUMOylating enzyme, associated with the INM, resulted in nuclear shape change and also affected distribution of nups. Loss of one of the proteins, namely Uip4, compromised the nuclear permeability barrier. We show that Uip4 localizes predominantly to the NE/ER and loss of Uip4 results in dramatic mis-localization of the NPCs. Furthermore, we also find that Uip4 expression is increased when the cells transition to stationary phase of growth. Despite the knowledge of basic structural components of NE and NPCs, a clear understanding of the underlying mechanisms that contribute toward the maintenance of shape of NE and integrity of associated complexes is lacking. This work opens up avenues for understanding multiple ways in which cellular components can contribute towards the maintenance of nuclear shape, integrity, and thereby function.

## Materials and methods

### Yeast strains and growth

The yeast strains used in this study are listed in Table 1. All strains were grown either in YPD (1% yeast extract, 2% peptone, and 2% dextrose) or SC (0.75% Synthetic Complete dropout powder mix prepared using yeast nitrogen base containing ammonium sulfate, 2% dextrose) media. Yeast transformation was performed using standard lithium acetate-based protocol (Daniel Gietz and Woods,)[30]. For all experiments, yeast strains were grown to mid-log phase at 30°C. For stationary phase, cells were harvested after 36 hours of growth. C-terminal 13MYC tag at the genomic loci of *UIP4* and *ESC1* was introduced by PCR based homologous recombination using pFA6a-13MYC-HIS3MX6 as described in [31]. *UIP4* was tagged with GFP at its C-terminal similarly using pYM25 (Janke et al., [32]). The high-efficiency transformation of PCR product for deletion and tagging was performed as described [33].

**Table 1.** List of strains used in the study

Name	Genotype/ Description	Source
KRY0437	*MATα ade2-1 leu2–3 − 112 his3-11,15 trp1-1 ura3-1 (W303B)*	Thomas and Rothstein, [34]
KRY1492	*MATa his3Δ1; leu2Δ0; met15Δ0; ura3Δ (BY4741)*	Euroscarf
KRY1493	*MATα his3Δ1; leu2Δ0; lys2Δ0; ura3Δ [BY4742)*	Euroscarf
KRY1570	*MATa KRY1492* except *UIP4-13MYC:HIS3*	This study
KRY1648	*MATa* KRY1654 except *uip4::KanMX*	This study
KRY1652	*MATα NUP188-GFP::HIS3*	[19]
KRY1653	*MATα NUP157-GFP::HIS3*	[19]
KRY1654	*MATa POM33-GFP::HIS3*	[19]
KRY1655	*MATa* KRY1652 except *uip4::KanMX*	This study
KRY1656	*MATa* KRY1653 except *uip4::KanMX*	This study
KRY1697	*MATa* KRY1492 except *uip4::KanMX*	Euroscarf
KRY1698	*MATα* KRY1493 except *uip4::KanMX*	Euroscarf
KRY1699	*MATa* KRY1492 except *uip3::KanMX*	Euroscarf
KRY1758	*MATa* KRY1570 except *nup133::KanMX*	This study
KRY1917	*MATa* KRY1492 except *uip1::KanMX*	Euroscarf
KRY1947	*MATa* KRY1492 except *uip2::KanMX*	Euroscarf
KRY1948	*MATa* KRY1492 except *uip5::KanMX*	Euroscarf
KRY 1950	*MATa UIP4-GFP-HYG^R^ DsRed-HDEL-TRP1*	This study
KRY1577	*MATa* KRY1493 except *pom34::KanMX uip4::KanMX*	This study
KRY1679	*MATa* KRY1493 except *nup157::KanMX uip4::KanMX*	This study

For assaying growth by spotting on solid media, equal number of cells from overnight cultures were sub-cultured in to 5 ml of fresh medium. Each of them was allowed to grow in the liquid synthetic complete medium for approximately 4 hours at 30°C. The cells were spun and a 10-fold serial dilution was performed. 5 µl of cells were spotted on SC plates and incubated for 2–3 days at desired temperature to assess growth differences. For growth curve analysis in liquid media, overnight cultures were sub-cultured to 200 µl volume in a 96 well plate. Biological triplicates for each strain were used. The OD600 was measured and cells were diluted to an initial OD600 of 0.1–0.12. The plate was incubated at 30°C with mild shaking. OD600 was recorded using a multimode plate reader every 90–120 min and growth curve was plotted. The readings were taken until the growth curve flattened and cells ceased to divide.

### Plasmids

All plasmids used in this study have been listed in Table 2. GFP-Esc1 [35], GFP-Nup49 [36], and NLS-2XGFP and NLS-2XGFP-NES [37] have been previously described. To generate pRS313-UIP4, the gene was amplified from genomic DNA of wild-type BY4741 strain with its own promoter (500 bp upstream of start) and 3ʹUTR [150 bp downstream of STOP) using Vent DNA Polymerase. For over expression of UIP4, the gene was amplified beginning from start codon upto 150 bp downstream of STOP. The fragment was inserted into the BamHI/SalI site of pBEVY-L under the *pGPD* promoter. Uip4-13Myc was amplified using genomic DNA of KRY1570 and cloned into the XbaI/SalI site of pBEVY-L. All constructs were confirmed by sequencing.

**Table 2.** List of plasmids used in the study

Name	Description	Source	Reference
CKM032	pRS313	Addgene	Sikorsky & Hieter, 38
CKM067	pFA6a-KanMx	Mark S. Longtine	[31]
CKM074	pFA6a-13MYC-HIS3MX6	Mark S. Longtine	[31]
CKM353	pUN100-GFP-Nup49	V. Doye	Doye et.al. JCB 39
CKM361	GFP-Esc1-pDZ45 [*GFP inserted in the XbaI site at 1431bp of ESC1*]	Imlitoshi Jamir	[35]
CKM461	pRS426-PADH1-NLS-[P12]-GFP-NES	K. Weis	[37]
CKM462	pRS426-PADH1-NLS-[P12]-GFP	K. Weis	[37]
CKM464	*pGPD*-UIP4-*ADH1t (Full length UIP4 inserted in the BamHI and SalI of* pBEVY-T]	This Study	
CKM466	UIP4-pRS313 (*Full length UIP4 with 500 bp upstream of start and 150 bp downstream of STOP cloned in BamHI and SalI site)*	This Study	
CKM501	pGPD-UIP4-13MYC-pBEVY-L *(Full length UIP4 with a C-terminal 13MYC epitope cloned in XbaI and SalI site of pBEVY-L vector for constitutive overexpression)*	This Study	
CKM502	pRS316-Nup49-mCherry	V. Doye	Chadrin et.al. JCB 40
CKM506	*pGPD*-UIP4-*ADH1t (Full length UIP4 inserted in the BamHI and SalI of* pBEVY-L)	This Study	

### Live cell imaging

**Figure 1.**
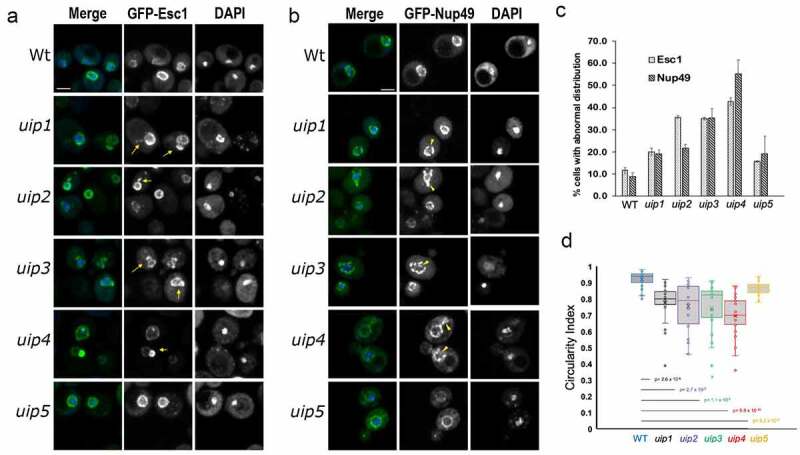
Loss of UIPs results in altered nuclear shape and nuclear import. **a, b**) Wt and strains harboring indicated deletions were examined for nuclear morphology and distribution of nuclear pores using GFP-Esc1 (a) and GFP-Nup49 (b) plasmids respectively. The maximum intensity projection (MIP) of representative cells is shown. DAPI staining is used to define the nucleus. The abnormality in the nuclear membrane and pore complex distribution is shown by yellow arrows and arrowheads respectively. Scale-2μm . **c)** Quantification of defects is shown in the bar graph as the fraction of cell population showing abnormality (n>300, 3 independent experiments, error bars indicate SEM). **d)**The circularity index of the nuclear envelope for the indicated strains is shown. 25-30 cells picked randomly were used for measurement. The horizontal line shows the mean of the distribution.

Cells grown were harvested by centrifugation, and the pellet was washed twice with fresh SC media. For images shown in Figure 1(a,b), Fig S1C and Fig S4G cells suspended in SC media were added to Concanavalin A-coated slides. The cells were allowed to settle for 5 minutes and excess liquid was removed using a vacuum pump. For nuclear staining, 5 µl of 50 ng/ml DAPI was added to the cells in dark. After 5 minutes, 4 µl of Slow Fade Anti-fade mounting medium (Invitrogen S36936) was added and coverslip placed. The edges were sealed gently using nail polish. For other live cell and time lapse imaging, cells resuspended in growth media were mounted onto a 35 mm cover glass bottom dish coated with ConA. After allowing the cells to settle, unbound cells were removed by rinsing with SC media. Images were acquired in Leica TCS SP8 equipped with a temperature controlled stage set at 30°C. Cycloheximide was added to a final concentration of 10 µg/ml in the imaging medium for experiment shown in Fig S3D.

### Immunofluorescence

Immunofluorescence was performed as described in [41]. Briefly, cells were fixed in 3.7% formaldehyde at 30°C for 20 minutes, followed by three washes with sterile distilled water. Cells were then treated with 10 mM DTT and 0.1 M EDTA-KOH for 10 minutes at 30°C. Spheroplasts were generated by treating the cells with 0.25 mg/ml Zymolyase 100 T at 30°C until the cell wall was digested. Digestion of cell wall was confirmed by visualizing a drop of cell suspension under a light microscope. Appropriate amount of spheroplasts were dropped onto a poly-L-Lys coated slides. The cells were permeabilized by treating with methanol and acetone for 5 minutes and 30 seconds, respectively. Blocking was done using 1% BSA prepared in 1X-PBS (pH 7.4) with 0.1% Tween-20. Primary antibodies α-Myc (Abcam ab9106) and α-Nsp1 (Abcam ab4641) were used at a dilution of 1:800, and α-Pdi1 (Abcam ab4644) at 1:500 and stained overnight at 4°C in a closed, moist slide box. Staining with secondary antibodies labeled with either AF488 (Invitrogen) or Cy3 (Jacksons) at a dilution of 1:1000 was done at room temperature for 2 hrs. DAPI staining was done for 5 minutes followed by mounting with Prolong gold antifade mountant (Invitrogen, P36930).

### Stimulated emission depletion (STED) microscopy

To achieve a higher resolution, we used STED-based confocal microscopy. Indirect immunofluorescence was performed for wild-type and *uip4∆* cells harvested in mid-log phase, as described above. Alexa Fluor 488 labeled secondary antibody was used to detect the Nsp1 signal. Excitation was carried out with 488 nm laser, and 592 nm depletion laser was used for resolution enhancement. DAPI was excluded since its emission spectra interferes with the depletion laser.

### Deconvolution

**Figure 2.**
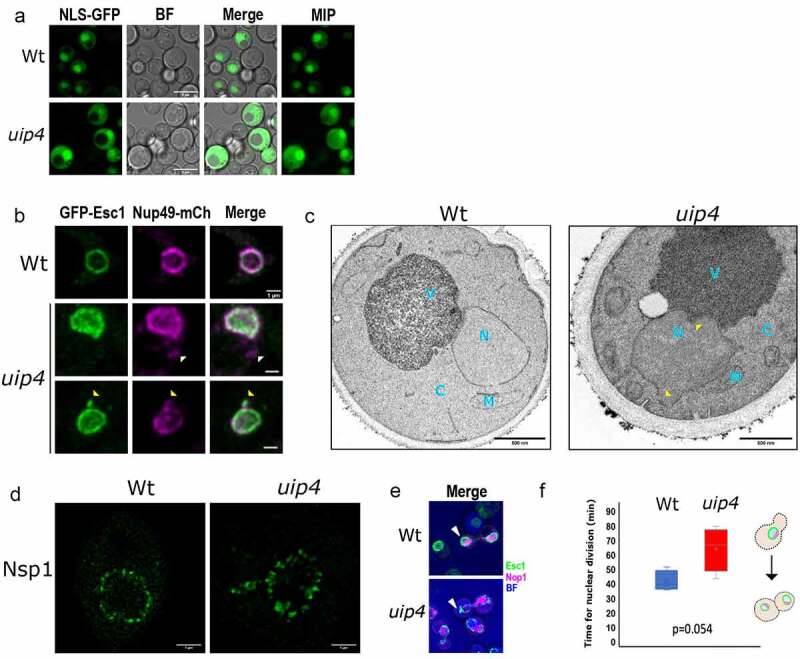
Loss of UIP4 results in altered nuclear shape and nuclear import. **a)**Nuclear import was tested in Wt and uip4 bearing NLS-2X GFP plasmid. Representative images are shown. Scale-5μm **b)**Representative images of Wt and uip4 co-transformed with GFP-Esc1 and Nup49-mCherry are shown. White arrowheads indicate non-NE associated nups and yellow arrowheads indicate NE extensions bearing nups. Scale-1μm **c)**Transmission electron micrographs for Wt and uip4 are shown. N-nucleus, C-cytoplasm, V-vacuole, M-mitochondria. yellow arrowhead- NE deformity in uip4. Scale-500nm **d)** Deconvolved STED micrographs for Wt and uip4 are shown. The signal in green shows Nsp1 localization.Scale-1μm **e)** MIP of dividing Wt and uip4 cells with NE (GFP-Esc1) in green and nucleolus (mRFP-Nop1) in magenta are shown. Arrowheads- associated NE abnormality. **f)** The plot indicates the time taken by Wt and uip4 cells to complete nuclear division. The horizontal line represents the mean.

Images shown in Figure 2(b,d) were deconvolved using the deconvolution wizard of Huygens Professional version 17.04 (Scientific Volume Imaging, The Netherlands, http://svi.nl). Confocal images were exported to Huygens Professional and an automatic estimation of the background was done using the in/near object estimation mode. GMLE (Good’s roughness Maximum Likelihood Estimation) deconvolution algorithm was used and images were deconvolved on the basis of point spread function (PSF) in an iterative fashion. The signal-to-noise ratio (SNR) was set between 10 and 14 depending on the intensity of fluorophore.

### Electron microscopy

For transmission electron microscopy, yeast cell cultures were grown in SC media and harvested at mid-log phase (0.8 OD at A600). The method was adapted from Levi et al., 2010. Briefly, cells were fixed in ice-cold fixing solution (2% glutaraldehyde and 1% paraformaldehyde, 1 mM MgCl2, in 50 mM potassium phosphate buffer (pH 6.8) for 2 hrs at 4°C, followed by post-fixation in 4% KMnO4 for 1 hr at room temperature (RT). Cells were treated with freshly prepared 2% uranyl acetate for 1 hr at RT and subsequently washed. Dehydration of cells in increasing ethanol concentration (50%–100%) was followed infiltration with the combination of 100% Araldite resin. Further, to enhance the contrast, sections were stained with lead citrate and micrographs were acquired on JEM-1400 (Japan) at 120kV.

### Western blotting

Cells transformed with desired plasmid were grown in selection medium either upto mid-log phase or stationary phase, as desired. Proteins were extracted using TCA method of protein precipitation [42] Briefly, 200 µl of 20% TCA was added to the cell pellet and was lysed by adding glass beads and vortexing at high speed for 3 minutes. The lysate was transferred to a fresh 1.5 ml tube. The tube containing glass beads was washed twice with 200 µl of 5% TCA and vortexing for 1 minute. The lysate was collected and added to the previous tube. The entire lysate was spun at 13000 rpm for 10 minutes. Supernatant was discarded completely, and the pellet was dissolved in 200 µl of Laemmli buffer. 2 M Tris was added dropwise until the buffer turned blue. Proteins were denatured by boiling in Laemmli buffer for 5 minutes at 95°C. SDS-PAGE was done followed by a standard semi-dry method of transfer to PVDF membrane for Western blotting. Primary anti-Myc (Abcam -ab56, 1:10000), anti-GFP (Abcam ab290, 1:3000), anti-Nsp1 (Abcam ab4641, 1:10000), and anti-actin (Santa Cruz sc-47778, 1:5000) were detected by HRP tagged secondary anti-rabbit (Abcam ab97051) and anti-mouse (Santa Cruz sc-2005). The signal was detected by ECL reagent (GBiosciences) and imaged in ChemiDoc Imaging system by BioRad.

### Quantification

Quantification of the phenotype for all presented data was done from at least three independent experiments. At least 100 cells were analyzed from each experiment. Representative images are shown. Bar graphs represent the fraction of population showing the indicated phenotype. Where shown, error bars represent SEM. p-values to assess significance was determined by Student’s t test. In a microscopy-based approach, the differences can be inspected visually. Such changes were scored manually and reported as the fraction of cell population that has different morphology as compared to the wild-type cells or the untreated cells. NPC aggregation index was determined as described in [25]. Briefly, the NE outline based on the signal from indicated nup was traced in FIJI using the freehand tool. The selection was converted to a line, and fluorescence intensity values were obtained using the plot profile function. The intensity values for around 20–25 cells from at least two independent experiments were copied to excel sheet, and respective background fluorescence intensity was subtracted. Next, for each NE, average intensity was subtracted from the individual values and the absolute difference was obtained. The standard deviation of this difference was divided by the average intensity of each NE and referred to as the aggregation index.

## Results

### Loss of Ulp1 interacting proteins results in distorted nuclear morphology

To identify the components that contribute to the process of regulating nuclear shape, we initiated a fluorescence microscopy-based screen [17, 35 and unpublished data]. To this end, non-essential gene knockout strains of BY4741 background were systematically transformed with a GFP-tagged inner nuclear membrane protein (ScEsc1). We have previously validated the use of GFP-Esc1 as a marker for nuclear shape in budding yeast [35]. Wild-type cells display a round, nuclear envelope staining (Figure 1a, row 1), but several gene knockouts identified in the screen have abnormal nuclear shape [17,35]. The phenotype is primarily scored as the fraction of cell population displaying deviation in nuclear morphology as compared to the wild type. The mutants in which more than 15% of the population shows abnormality are further assessed using other markers for determining NE morphology and the circularity index is determined to evaluate the abnormality further. Our results from the genome-wide screen show that around 10% of the non-essential deletion mutants analyzed have distorted nuclear morphology (unpublished data). Such mutants belong to diverse functional categories and have various kinds of NE abnormalities as visualized by either GFP-Esc1 and/or GFP-Nup49 distribution.

While screening the non-essential gene knockout collection of *S. cerevisiae* for nuclear shape in a chromosome-wise manner, we found that loss of YAL014C (*UIP2)* and YAR027W (*UIP3)* from chromosome I resulted in altered nuclear shape (Figure 1a). Extensions of the NE and irregular shape was observed in about 35% of the *UIP2* and *UIP3* knockout cells (Figure 1(a–c)). *UIP2* and *UIP3* were earlier identified as interactors of Ulp1p in a yeast two-hybrid screen [43]. ScUlp1 is a SUMO protease, which localizes to the nuclear periphery and associates with the NPCs (Li and Hochstrasser, [44]; Schwienhorst et al., [45]), and its function is essential for maintaining NPC homeostasis [Li and Hochstrasser, 1999; 46]. Six proteins, Uip1 through Uip6, were identified in the two-hybrid screen, and two of those showed nuclear morphology changes. Uip1p and Uip6p are established components of NPCs in yeast; Uip1p is a non-essential FG nucleoporin- Nup42 and Uip6p is an essential cytoplasmic Nup- Gle1 [5]; Uip2 is a syntaxin (Syn8); the other Uips have not been characterized so far. We examined the deletion mutants of Uip1 to Uip5 for nuclear shape defects (Fig S1A). We did not test Uip6 as it is an essential gene.

The shape of the nucleus as marked by GFP-Esc1 was severely distorted upon loss of *UIP3* and *UIP4* (Figure 1a -arrows, 1c). Clear membrane extensions and flares could be seen upon loss of *UIP*2. *uip3*∆ and *uip4*∆ cells had large membrane blebs. Previous studies have reported an intricate dependence of nuclear shape and NE dynamics on the distribution of NPCs [47]. Mutants that have aberrant NPC distribution are often accompanied by nuclear shape abnormalities. Also, Esc1 is known to modulate the Ulp1 distribution and nuclear basket assembly at the NE [48]. Therefore, we wanted to see if the NPC distribution is affected upon loss of these *UIPs*. GFP-Nup49 was used to examine the distribution of NPCs. We found that loss of *UIPs* resulted in clustering of Nup49 as compared to its distribution in wild-type cells (Figure 1b -arrow heads, 1c). This clustering phenotype was moderate in *uip1∆, uip2∆,* and *uip5∆* while prominent in a large fraction of *uip3∆* and *uip4∆* cells (Figure 1c). The nuclear shape abnormalities were also evaluated by measuring the circulating index (CI) of the nuclei in the deletion mutants (Figure 1d). We find a wide variation in the nuclear morphology in the deletion mutants. A large fraction of *uip4∆* cells had severe nuclear shape abnormalities as well. Blebs and extensions of nuclear membrane was the most prominent nuclear shape defect in *uip4*∆. The nuclei of *uip5∆* cells were mostly circular and the other *UIP*s had moderate levels of distortions (Figure 1d, summarized in Fig S1A). Despite compromised nuclear structure, none of the deletion mutants had any growth defects (Fig S1B).

### Uip4 is required for maintaining nuclear structure and function

NE serves as a selective barrier between cytoplasm and nucleoplasm. To check the integrity of NE and functional competence of the nuclear pores, we tested the nuclear import capability of cells lacking UIPs using NLS-GFP construct [37]. Wild-type cells accumulate NLS-GFP in the nucleus efficiently as evidenced by a bright nuclear fluorescence (Figure 2a, S1C). Similarly, *uip1∆, uip2∆, uip3∆,* and *uip5∆* cells also import NLS-GFP in to the nucleus (Fig S1C). However, loss of *UIP4* showed a large defect in the nuclear import of NLS-GFP with about 40% of cells showing partial to complete exclusion of NLS-GFP from the nucleus (Figure 2a, S1C). This indicates a compromised nuclear permeability barrier upon loss of Uip4 and suggests that the clustered pores in other UIPs are functionally competent for nuclear import unlike in *uip4∆*. We also tested the distribution of NLS-NES bearing construct fused with GFP in the wild-type and *uip4∆* cells (Fig S2A). In the wild-type cells, the NLS-NES fusion protein is excluded from the nuclear region entirely in over 90% of the cell population when visualized in the plane of maximum nuclear diameter (inset Fig S2A, row 1). However, in the *uip4∆* cells, GFP is seen in nucleus as well as cytosol. Additionally, in ~29% of the population, GFP fusion protein is retained within the nucleus, indicating deficiency in nuclear export as well (Fig S2A, row 3).

Among the 5 UIPs studied for nuclear defects, loss of *UIP4* resulted in the most severe phenotype of a compromised nuclear structure and function. Therefore, we examined the effects of the loss of Uip4 further. We acquired images of cells expressing both GFP-Esc1 and Nup49-mCherry (Figure 2b). In *uip4∆,* most of the nuclei have irregular NE morphology and some of the cells have non-NE associated Nup49 signal (Figure 2b, row 2-white arrow head). Fluorescence micrographs with overlapping signal from the two markers indicate that some of the NE extensions (GFP-Esc1) bear nucleoporins (Nup49-mCherry), as shown by yellow arrow heads in Figure 2b. The irregular morphology of the NE was also confirmed by transmission electron microscopy (TEM). For wild type, most of the cells had quasi-round nuclear shape as observed based on the NE contour (Figure 2c, Fig S2B). However, the NE outline of a majority of *uip4∆* cells was distorted, as shown in Figure 2c (yellow arrowheads). Various abnormalities as seen in live cell imaging were also mirrored in the electron micrographs (Fig S2B).

To examine the distribution of nucleoporins along the nuclear periphery in *uip4*∆, we investigated the effect of loss of *UIP4* more carefully without using a plasmid-based fluorescent marker. We performed indirect immunofluorescence and stained the NPCs in wild-type and *uip4∆* cells using antibody to Nsp1-a cytoplasmic FG nucleoporin. Then, we acquired the images using Stimulated Emission Depletion (STED)-based super resolution microscopy (Hell and Wichmann,)[49]. STED involves use of an additional laser beam other than the excitation laser. The high intensity and high wavelength STED laser beam has a donut shape, which enhances the lateral and axial resolution of biological samples by turning the fluorophores outside of the diffraction limited spot to an ‘off’ state. Therefore, the fluorescent signal can be seen as clearer, resolved spots, rather than a contiguous blur (compare Figure 2b-d, Fig S2C). In wild-type cells, the NPCs are known to distribute along the nuclear periphery almost evenly as seen in the STED micrograph of wild-type cells in Figure 2d. This distribution becomes uneven (clustered), and non-nuclear as well, in the *uip4∆* cells (Figure 2d, right). Together, our observations strongly suggest that loss of *UIP4* results in nuclear shape distortion and affects distribution of nucleoporins resulting in clustering of NPCs.

Defective nuclear shape has been associated with delayed mitosis [50]. Therefore, we sought to monitor the nuclear shape dynamics during cell division by live cell microscopy. Cells were grown in a 35 mm cover glass bottom dish in the presence of SC medium at 30°C. *S. cerevisiae* undergoes closed mitosis, therefore, in wild-type cells the nuclei remained mostly round-elliptical with a uniform distribution of Esc1p around the nuclear periphery (Figure 2e, Fig S2D). However, *uip4∆* display a wide range of nuclear morphologies during nuclear transmission to the bud (Figure 2e, Fig S2D, arrow heads). This resulted in an increased nuclear division time for *uip4∆* as compared to the wild type (Figure 2f). However, the overall growth rate for wild type and *uip4∆* was similar (Fig S2E).

### Loss of Uip4 results in abnormal NPC distribution

**Figure 3.**
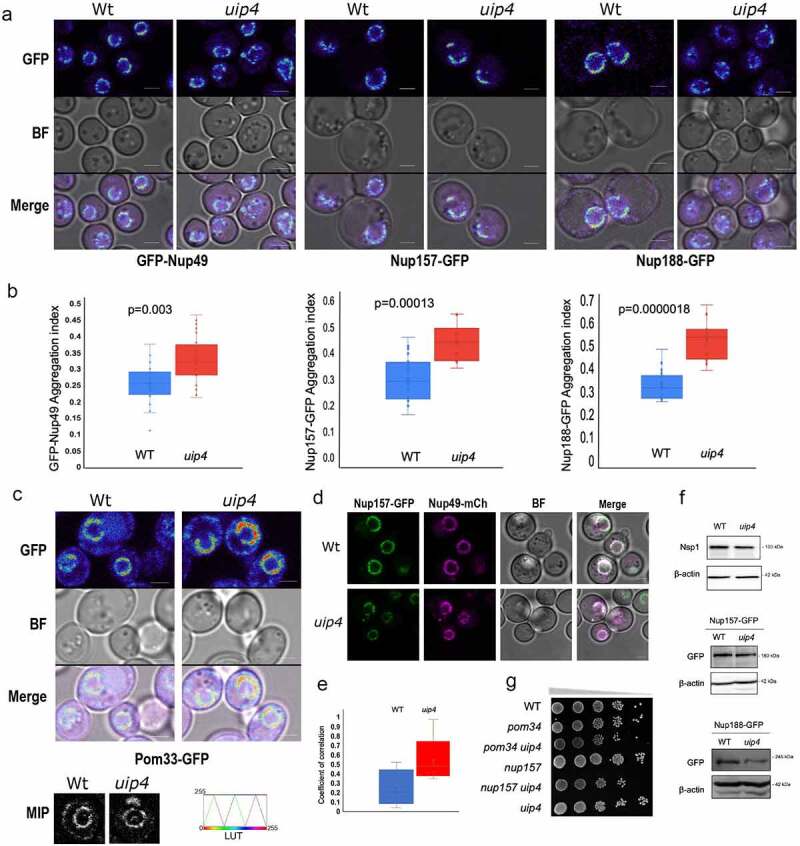
Loss of UIP4 results in NPC clustering **a)** Live cell imaging was done for Wt and uip4 strains expressing indicated GFP tagged Nups from their endogenous loci. The spectrum lookup table (LUT) is used to represent the GFP signal. Scale-2μm **b)** The box plot represents the aggregation index calculated from the GFP signal along the NE in the mid-focal plane of 25-30 individual cells from Wt (blue) and uip4 (red). The horizontal line is the mean value. **c)** Live cell imaging was done for Wt and uip4 strains expressing Pom33-GFP from its endogenous loci. Spectrum LUT is used for the GFP signal. Scale-2μm The inset shows MIP of a representative nucleus from Wt and uip4 in grayscale. The scale for Spectrum LUT is shown on the right. **d)** Fluorescence micrographs for Wt and uip4 co-expressing Nup157-GFP and Nup49-mCherry are shown. Scale-2μm. **e)** The extent of co-localization for Nup157-GFP and Nup49-mCherry shown in D was assessed by measuring the correlation coefficient between the two signals in the non-NE region. The horizontal line represents the mean. **f)** Western blot analysis was done to compare the expression levels of Nsp1, Nup157, and Nup188 in Wt and uip4 cells harvested from the mid-log phase. α-GFP was used to detect endogenously tagged Nup157 and Nup188, and actin is the loading control. **g)** Overnight cultures of the indicated strains were taken and sub-cultured by inoculating an equal number of cells in a fresh medium for 4 hours. Cells were then harvested and 10-fold dilutions were serially spotted on an SC plate. The plates were incubated at 30ºC for 2 days prior to imaging.

Proper NPC assembly and distribution is crucial for maintenance of the nuclear shape [21,51]. While in wild-type cells, less than 10% cells had nuclei that were not spherical, in *uip4*∆, over 50% of cells had distorted nuclear shapes and clustered nuclear pores as visualized by distribution of Nup49 (Figure 1c). Similar clustering along the nuclear periphery was also seen for Nup84-mcherry in *uip4∆* cells (Fig S2A). Nup84 is a subunit of the yeast Nup84-subcomplex and is involved in nucleocytoplasmic transport (Siniossoglou et al., [52]). Yeast Nup84 is homologous to mammalian Nup107, which is required for proper NPC assembly (Boehmer et al.,)[53]. We then examined the distribution of inner ring nucleoporins namely Nup157 and Nup188. Both of these nups were also clustered upon loss of *UIP4* (Figure 3a,b). We quantified the clustering of NPCs by calculating the aggregation index based on the distribution of fluorescence signal intensity of GFP tagged nucleoporins along the NE. The aggregation index for GFP-Nup49 in *uip4∆* cells was found to be significantly higher than that in wild-type cells (Figure 3a,b). The transmembrane nup, Pom33, showed a differential localization in the *uip4∆* cells. A minor fraction of Pom33 is localized to the ER in addition to the NE in wild-type cells (Chadrin et al., [54]). Apart from clustering at the NE, there was a reduction in the distribution of Pom33 to the cortical ER in *uip4∆* as compared to wild-type cells, with most of Pom33 present in the NE (Figure 3c). The maximum intensity projection (MIP) of a single representative cell from wild type and *uip4∆* is shown for comparison (Figure 3c, lower left). These observations were also reiterated by indirect immunofluorescence using GFP antibody to detect Nup157, Nup188, and Pom33, and co-stained with anti-Nsp1 (Fig S3A). Loss of Uip4, therefore, seems to affect distribution of multiple nups.

Apart from aggregation of NPCs at the NE, we also observed presence of cytosolic spots of nups in *uip4∆* (Figure 3d). Although cytosolic pool of nucleoporins in wild-type cells has been reported earlier [24,26,27], this fraction of cell population was at least three-fold higher in *uip4∆* cells (Fig S3B). Complementation with a plasmid borne copy of *UIP4* expressed from endogenous promoter could rescue the phenotype in *uip4∆* (Fig S3B). We assessed the relative distribution of nucleoporins from two sub-complexes between wild-type cells and cells lacking UIP4. To do so, we analyzed the degree of co-occurrence between two nups by quantifying coefficient of correlation derived from the intensity of fluorescence signal. The NPC clusters at the nuclear periphery do not differ in their relative nup constitution between wild type and *uip4∆* as determined based on the degree of colocalization of Nup157 and Nup49 (Figure 3d, S3C). Contrary to this, the cytosolic spots observed in *uip4∆* contain more than one nup unlike in wild type (Figure 3e, S3C).

The cytosolic foci observed in *uip4∆* could be either assembled NPC assembly intermediates or disintegrated NPCs. To delineate if the cytosolic signal is indicative of *de novo* NPC assembly intermediates, we treated wild-type and *uip4∆* cells with cycloheximide, a protein translation inhibitor [John J. 14, 25]. If the cytosolic spots are due to disintegration of NPCs, they should continue to increase even in the absence of fresh protein synthesis. We monitored GFP-Nup49 in wild-type and *uip4∆* cells by time-lapse live cell imaging after addition of cycloheximide (Fig S3D). We did not see any increase in the cytosolic foci indicating that these cytosolic spots are intermediates of NPC assembly (Fig S3D). Interestingly, we also observed that after two hours of cycloheximide exposure, even in wild-type cells, the NPCs began to cluster and nuclear morphology also began to distort (Fig S3D, S3E). The effect of loss of *UIP4* on the stability and turnover of individual nucleoporins warrants further investigation. Our western blot results indicate that the total protein levels for Nsp1 and Nup157 do not differ much between wild type and the *uip4∆,* and the modest reduction seen in Nup188 is also statistically insignificant (Figure 3f), again indicating that these are unlikely to be disintegrating NPCs.

Since loss of *UIP4* compromised function of NPCs, we wanted to test if Uip4 interacts with nucleoporins or other NE proteins that contribute to NPC stability. To see if there is a genetic interaction, we created double mutants with POM34 and NUP157. Pom34 is one of the three primary transmembrane nup in yeast [55]. Pom34 is important during the NPC biogenesis, whereas Nup157 is required for ensuring proper assembly of pores at later stages [56]. We did not observe a strong genetic interaction of *UIP4* with these nups either in spot assays on plate (Figure 2g) or liquid growth cultures (Fig S3F). Furthermore, we attempted to test if the effect of Uip4 on NPC function is due to its interaction with Ulp1 [46,43]. Although Uip4 was originally identified in a yeast two-hybrid screen for interactors of Ulp1, we did not find any positive interaction between Ulp1 and Uip4 by direct two-hybrid (data not shown). Additionally, in a yeast two-hybrid screen to obtain other physical interactors of Uip4, we did not detect any nucleoporin or nuclear protein that is known to directly affect NPC function. Taken together, these results indicate that although the presence of Uip4 contributes positively toward NPC assembly and stability, this effect is likely to be a consequence of either a transient association of Uip4 with nups or an indirect association.

### Uip4 localizes to NE/ER

The biological function of Uip4 is not known so far. To get some insight into the function of the protein, we began by identifying domains or sequence-based features of the protein. We searched for conserved domains within the protein sequence using Conserved Domain Database (CDD) search [57]. Uip4 harbors an N terminal (1–87aa) Early-set glycogen binding domain (E-set GBD) and a MDN1 superfamily midasin domain (73–271aa), as depicted in Fig S4A. The Early-set GBD has an Ig fold like beta-sheet structure and is known to bind glycogen (Polekhina et al., [58]). The remainder of the protein shows similarity to the linker region of MDN1 in *Saccharomyces cerevisiae* and is rich in aspartate and glutamate residues (Garbarino and Gibbons, [59]). It lacks a transmembrane membrane domain but has a KKXX (_277_KKLL_280_) motif, suggesting potential ER membrane retention. The amphipathic helix at the C-terminal end of Uip4 is likely to act as its in-plane membrane anchor (predicted by AMPHIPASEEK-Sapay et. al).

**Figure 4.**
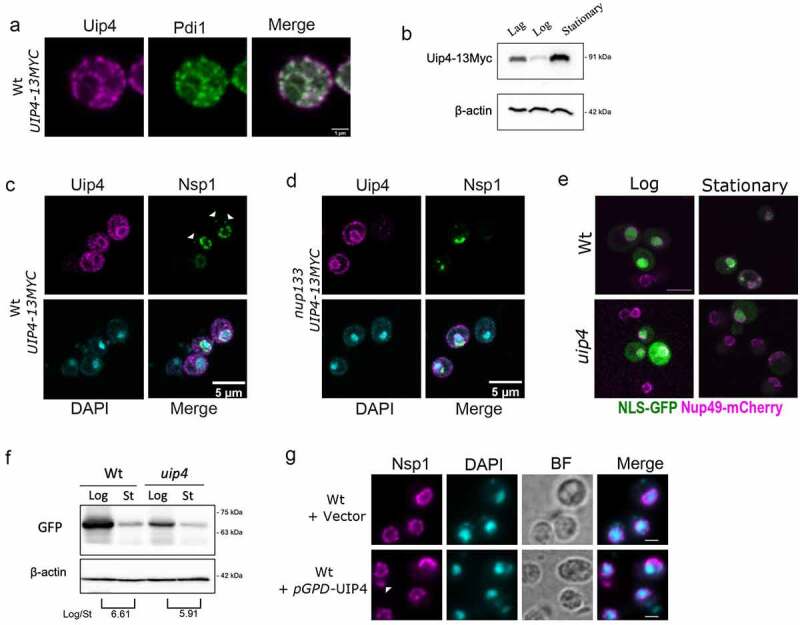
Uip4 localizes to NE/ER and its overexpression exacerbated NE defects **a)** Indirect immunofluorescence using α-myc was performed in the strain encoding UIP4-13xMyc to check the localization of Uip4. Co-staining with an ER marker Pdi1 is shown. Scale-1μm **b)** The western blot is representative of the expression level of Uip4 in cells harvested from either lag (O.D.600- 0.2-0.4), mid-log (O.D.600- 0.8-1.2), or stationary phase (O.D.600- 3.0-3.5). Uip4 was detected using α-myc. Actin is used as a loading control. **c)** Co-staining of Uip4 with an NPC marker, Nsp1, was done in cells harvested from the stationary phase. White arrowheads indicate non-NE associated cytosolic spots. Scale-5μm **d)** Localization of Uip4 in nup133 background was checked. Co-staining of Uip4 with an NPC marker, Nsp1, is shown. Scale-5μm **e)** Live cell imaging was performed in Wt and uip4 cells bearing NLS-GFP plasmid harvested from either mid-log or stationary phase. The overlay images showing NLS-GFP (green) and Nup49-mCherry (magenta) are indicative of the observation. Scale-5μm **f)** Western blot showing protein level of NLS-GFP detected using α-GFP is shown for wild type and uip4 cells harvested from log and stationary (St) phase. The quantification of the NLS-GFP in the Log/Stationary phase is shown (Average of 2 independent experiments). Actin is used as a loading control. **g)** The distribution of NPCs upon UIP4 OE was checked by monitoring the localization of Nsp1, an FG nucleoporin. Indirect immunofluorescence was performed in WT cells transformed with either E-pBevyL or pGPD-UIP4-pBevyL. White arrowhead indicates non-nuclear spots of Nsp1. Scale-2μm

To study the expression and subcellular localization of Uip4p, we tagged it with a C-terminal GFP tag (Janke et al., 2004). Because of low expression of Uip4p and photobleaching effect in confocal microscopy, GFP did not result in a good signal to noise ratio of the images (Fig S4B). To overcome this, we tagged *UIP4* with a C-terminal *13X-MYC* epitope at the endogenous loci [31] and indirect immunofluorescence was done in cells expressing Uip4-13xMyc. Using secondary antibody conjugated to an organic fluorophore resulted in a brighter and stable fluorescence. Tagging Uip4 did not cause a loss of function as the nuclear shape and NPC distribution in strain expressing 13xMyc tagged UIP4 was similar to that of wild-type cells (Fig S4C). To test the localization of Uip4, we performed indirect immunofluorescence using anti-myc antibody and co-stained the cells with Pdi1 as an ER marker. In consonance with the presence of KKLL motif, indirect immunofluorescence results showed that Uip4 localizes to two prominent rings resembling ER distribution, in addition to some cytosolic signal (Figure 4a, Fig S4D). The distribution of Uip4-13xMyc largely overlapped with Nsp1 at the perinuclear ER suggesting that Uip4p is an NE/ER protein (Fig S4D). Expression of the protein was confirmed by Western blot analysis (Figure 4b). We observed a higher expression of Uip4 in cells harvested from stationary phase as compared to those in mid-log phase (Figure 4b). The Uip4p localization was largely unaffected during stationary phase (Figure 4c), although a more continuous staining of Uip4 was present at the NE/ER. Wild-type cultures from stationary phase of growth also have cytosolic presence of nups that display foci like distribution distinct from the NE (Figure 4c, arrow). The Uip4 signal did not overlap with the cytosolic spot of Nsp1. To test whether Uip4p localization is affected by strong clustering of NPCs, we tested the localization of Uip4p in *nup133∆*, a strain which is known to have clustered NPCs [60]. We found that in *nup133*∆, Uip4 was localized extensively to ER (Figure 4d). Even in this case, the two signals do not co-localize, suggesting that pore clustering does not influence Uip4p localization and Uip4p does not physically associate with NPCs.

### Regulated Uip4 expression is critical for NPC function

We found the Uip4p levels to be lowest during log phase when the cells are actively dividing and highest during the stationary phase when the growth is ceased (Figure 4b). This expression correlated with the higher defect in NPC distribution in *uip4∆* cells during stationary phase (Fig S4E). Similar to the cells from log phase culture, we observed more cells showing cytosolic foci of Nup49 in stationary phase as well, for *uip4∆*. This indicates that Uip4 could be important for NPC distribution particularly during stationary phase. To assess this further, we looked at the NE integrity by import of NLS-GFP (Figure 4e). Fluorescence imaging indicated an overall reduction in the signal intensity of NLS-GFP for cells harvested during stationary phase for both wild type and *uip4∆* (Figure 4e). Western blots also confirmed the lower level of NLS-GFP expression in both cells harvested from stationary phase as compared to those harvested in log phase (Figure 4f). In addition to defective nuclear import, in *uip4∆* cells the expression level of NLS-GFP was lower than respective wild type in all stages of growth (Figure 2a, Figure 4e,f). Importantly, in the stationary phase, the reduced NLS-GFP signal was almost entirely nuclear in wild-type cells. But in *uip4∆,* the NLS-GFP signal was either from dead cells or whole cells, and hardly any cells showed nuclear accumulation, indicating a more compromised NPC function.

Having established the physiological conditions that regulate Uip4 expression, we tested the NPC distribution upon ectopic overexpression of Uip4. In addition to the traditional loss-of function or deletion studies, mutant phenotype can be a result of overexpression or misexpression of the wild-type gene. This provides an alternative yet powerful tool to identify pathway components that might remain undetected otherwise (Prelich, [61]). We confirmed the overexpression of Uip4-13Myc by Western blot and then assessed the growth of cells over producing Uip4 (Fig S4F, Fig S2E). Overexpression of Uip4 did not have any negative effect on the mitotic division as compared to the wild type and *uip4∆* (Fig S2E). We overexpressed Uip4 from a strong promoter and observed severe clustering of Nsp1 at the NE (Figure 4g). Cytoplasmic spots were also observed upon Uip4 overexpression (Figure 4g). Nuclear accumulation of NLS-GFP was also greatly reduced suggesting that the nuclear import is severely defective when Uip4 is overexpressed (Fig S4G). We found that the NPC localization and NE integrity defects were exacerbated when Uip4 was overproduced. As both loss and overproduction of Uip4 led to distorted nuclear envelope architecture and defective protein import function, it appears that the levels of Uip4p are important for regulating its function at the nuclear envelope. Taken together, these results suggest that the dosage and distribution of Uip4 is critical for its function in budding yeast.

## Summary

We have identified a novel role of a previously uncharacterized protein- Uip4 which localizes to NE/ER. While a comparative sequence analysis approach has shown that Uip4 is an ascomycete specific protein [62]; proteins with E-Set domain are known in all eukaryotes. Therefore other organisms could have functional homologs of Uip4 but could not be identified by sequence comparisons. We find that loss of Uip4 leads to assembly of functionally compromised NPCs. It is likely that altered Uip4p regulation affects the quality of NE by causing dysregulation of NPC stoichiometry. Uip4 could be involved in stabilizing the assembly intermediates of NPC or the entire complex itself at the NE by virtue of its membrane association or via the amphipathic helix at the C-terminal end of Uip4. In the absence of *UIP4*, we speculate that the incorporation of these substrates into the NE/NPC is attenuated and therefore can be seen in cytosol.

Even though Uip4 was not found to be present physically at the clustered pores, our results show that presence of Uip4 is required for proper distribution of the pores. This dependence could be due to either a temporary association or an indirect interaction. Many proteins with other primary functions are known to affect NPCs due to their transient association with nups or dynamic localization to nucleus [11,14,63]. Since we find that Uip4 is overexpressed during stationary phase in wild-type cells, it would be interesting to study the molecular mechanism of Uip4 induced NPC aggregation. Compared to log phase cells, a larger fraction of Uip4 is recruited to NE in stationary phase and also in *nup133*∆ (compare Figure 4a–c). Under both these conditions, NPCs are clustered. However, whether any post translational modification of nucleoporins such as phosphorylation or ubiquitination facilitates such re-localization of Uip4 is a possibility that needs to be investigated [64,65].

The protein constituents of NPCs, which are among the longest lived protein complexes of the cell, undergo turnover [Daigle et al.,; 15,66, Toyama et al., [67]]. Regulation of starvation and aging-induced nucleoporin turnover is of particular interest in the field of neurodegenerative diseases. Recent work has contributed to our understanding of how NE integrity and NPC turnover is regulated under specific conditions such as either nitrogen or carbon starvation [68,69–74,75]. Such response is brought about by a coordinated action of players from multiple organelles. Since Uip4 bears a E-set domain that senses AMP (5`adenosine monophosphate) levels, it is likely to function in coordination with pathways involved in regulation of nutrient sensing to NE homeostasis. Why nuclear shape and NPC distribution is responsive to growth conditions is not known. However, the regulation of transcription at the nuclear periphery and association of mRNAs with the NPCs might have a role in this. Which components directly mediate the response to nutrient availability to the nuclear periphery and dictate shape alterations is an open question. Together, this study hints at the key role of a NE/ER protein in nuclear structure and function regulation under altered metabolic conditions. Future experiments will aim to identify the underlying pathways that control nuclear morphology and its components in response to such physiologically relevant signals.

## Supplementary Material

Supplemental Material

## Data Availability

The authors confirm that the data supporting the findings of this study are available within the article and its supplementary materials.
