# Supplementary material for: Uip4p modulates nuclear pore complex function in Saccharomyces cerevisiae
Source: Nucleus. 2022 Feb 16;13(1):79–93. doi: 10.1080/19491034.2022.2034286 (PMC8855845; doi:10.1080/19491034.2022.2034286)
Supplement: Supplemental Material [file KNCL_A_2034286_SM1817.zip › supplementary/s1 pdf.pdf]

Figure S1

A

Systematic Name	Yeast Gene Name	Mammalian Counterpart	Nuclear envelope defect	Other known function in yeast
YDR192C	UIP1, NUP42, RIP1	Human NUP42 can complement yeast mutant	Mild	NPC related functions
YAL014C	UIP2, SYN8, SLT2	STX8- Syntaxin 8	Moderate	Involved in Golgi to Vacuole transport
YAR027W	UIP3	None	Severe	Unknown
YPL186C	UIP4	None	Severe	Unknown
YKR044W	UIP5	LMAN2- Lectin mannose binding 2	Mild	Predicted to function in Golgi and ER organization

B

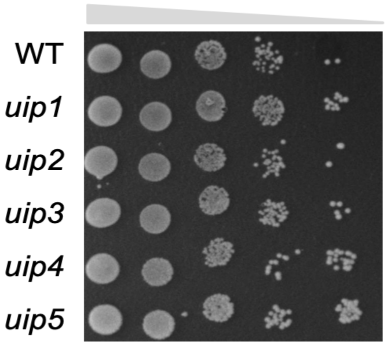

C

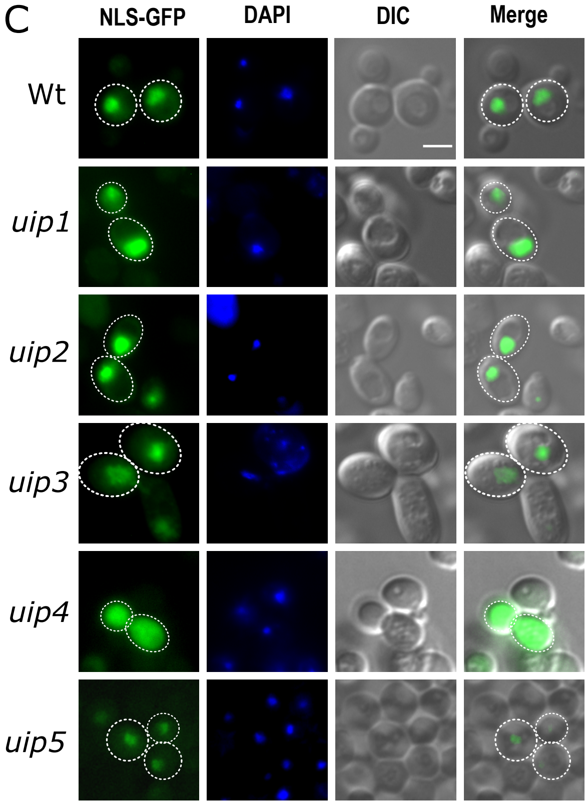

FigureS1.
A. Table with detail of UIPs screened for nuclear shape defect is presented. Information presented is sourced from Saccharomyces Genome Database.
B. In order to assess growth phenotype, overnight cultures of the indicated strains were taken and sub-cultured by inoculating an equal number of cells in a fresh medium for 4 hours. Cells were then harvested and 10-fold dilutions were serially spotted on a SC-plate. The plates were incubated at 30ºC for 2 days prior to imaging.
C. Nuclear import was tested in Wt and indicated strains bearing NLS-2X GFP plasmid. DAPI staining was used to define the nucleus. White dotted lines define the cell outline. Scale-2μm
