# Supplementary material for: Uip4p modulates nuclear pore complex function in Saccharomyces cerevisiae
Source: Nucleus. 2022 Feb 16;13(1):79–93. doi: 10.1080/19491034.2022.2034286 (PMC8855845; doi:10.1080/19491034.2022.2034286)
Supplement: Supplemental Material [file KNCL_A_2034286_SM1817.zip › supplementary/s2.pdf]

Figure S2

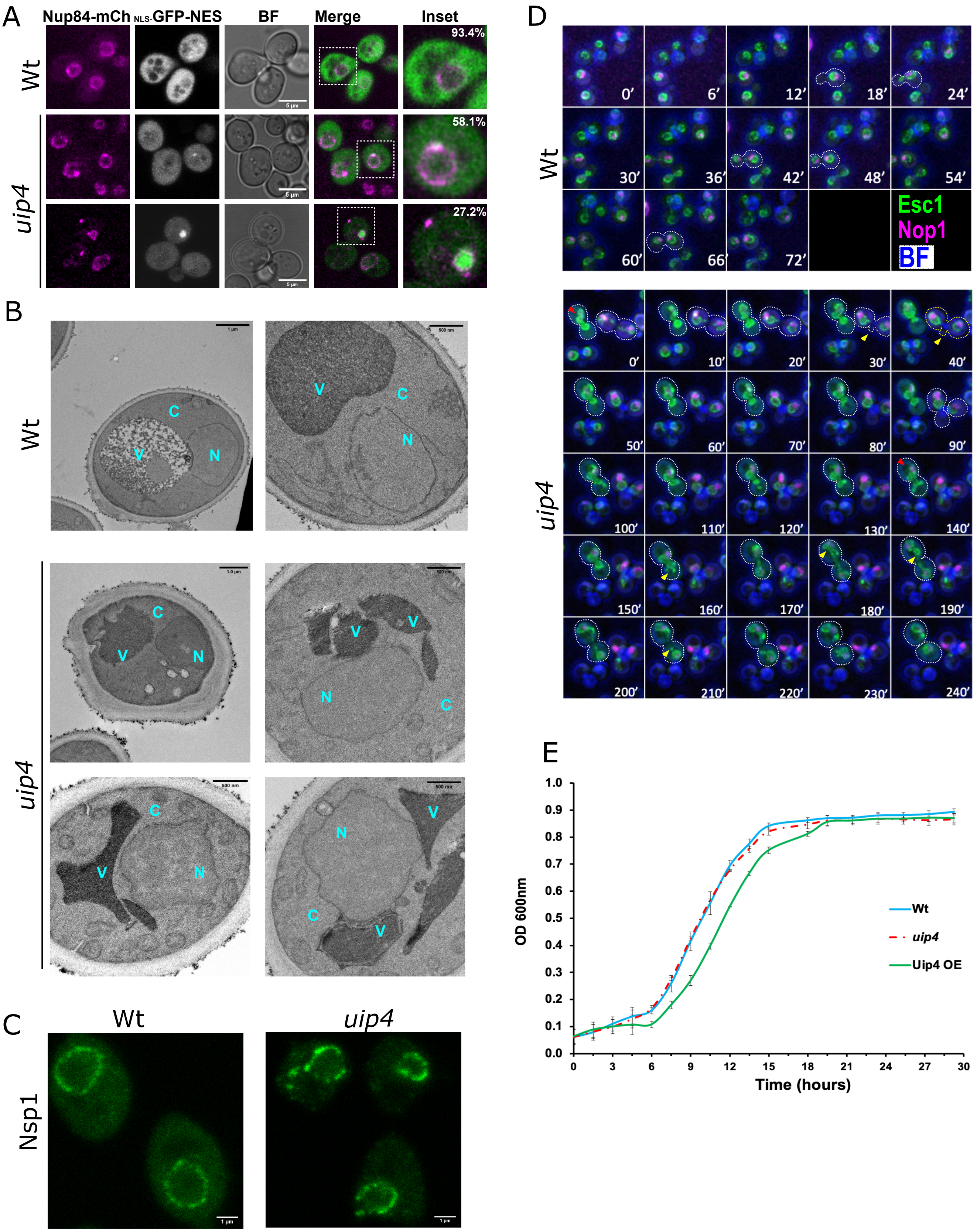

FigureS2.
A. Nuclear export was tested in Wt and *uip4* strains transformed with NLS-GFP-NES plasmid. The representative micrographs are shown. The strains were co transformed with Nup84-mCherry to define nuclear outline. Inset shows the cell outlined in the merge column. **B.** Additional TEM micrographs are shown for Wt and *uip4* strains. **C.** Confocal images acquired after STED are shown for Wt and *uip4*. **D.** Maximum intensity projections of Wt and *uip4* marking NE (GFP-Esc1) in green and nucleolus (mRFP-Nop1) in magenta acquired at indicated time points during time lapse live cell imaging are shown. Arrow heads- associated NE abnormality. **E.** Overnight cultures of the indicated strains were taken and sub-cultured into fresh medium at 30ºC. OD600 was recorded every 90 min and plotted. Three biological replicates of Wt (blue) and *uip4* (red) cells were used. Error bars represent SEM.
