# Supplementary material for: Uip4p modulates nuclear pore complex function in Saccharomyces cerevisiae
Source: Nucleus. 2022 Feb 16;13(1):79–93. doi: 10.1080/19491034.2022.2034286 (PMC8855845; doi:10.1080/19491034.2022.2034286)
Supplement: Supplemental Material [file KNCL_A_2034286_SM1817.zip › supplementary/s3.pdf]

Figure S3

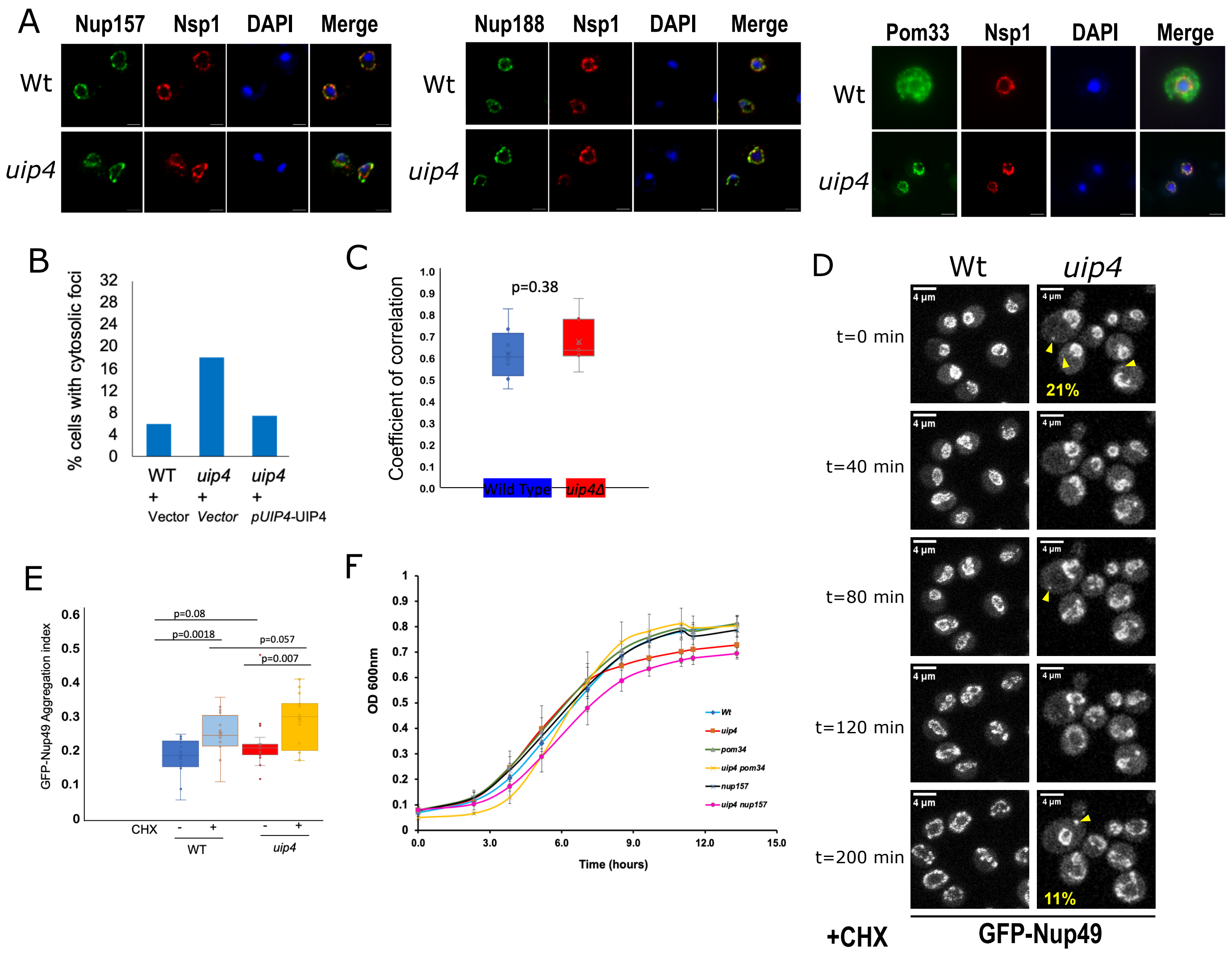

FigureS3.

A. Indirect immunofluorescence was performed in strains expressing either Nup157, Nup188 or Pom33 tagged with GFP. GFP and Nsp1 antibodies were used to detect respective nucleoporins. DAPI is used as a nuclear stain. Scale-2µm.

B. The bar graph represents the fraction of cells in the indicated strain complemented with either empty vector or vector with UIP4 cloned with its native promoter, showing cytosolic spots of GFP-Nup49. ~100 cells from 2 independent experiments were counted.

C. The extent of co-localization along the NE for Nup157-GFP and Nup49- mCherry shown in Fig2D was assessed by measuring the correlation coefficient between the two signals. Horizontal line represents the mean.

D. Maximum intensity projections of Wt and *uip4* expressing GFP-Nup49 acquired during time lapse live cell imaging are shown. Time indicates minutes after cycloheximide (CHX) addition. Yellow arrows show cytosolic spots and the quantification of fraction of cells is also indicated in yellow font. Scale-4µm

E.The box plot represents the aggregation index calculated from GFP signal along the NE in the mid-focal plane of 20-25 individual cells at t=0 (CHX -) and t=200min (CHX +) of cycloheximide addition. Horizontal line is the mean value.

F.Overnight cultures of the indicated strains were taken and sub-cultured into fresh medium at 30ºC. OD600 was recorded every 90 min and plotted. Three biological replicates of marked strains were used. Error bars represent SEM.
