# Supplementary material for: Uip4p modulates nuclear pore complex function in Saccharomyces cerevisiae
Source: Nucleus. 2022 Feb 16;13(1):79–93. doi: 10.1080/19491034.2022.2034286 (PMC8855845; doi:10.1080/19491034.2022.2034286)
Supplement: Supplemental Material [file KNCL_A_2034286_SM1817.zip › supplementary/s4.pdf]

Figure S4

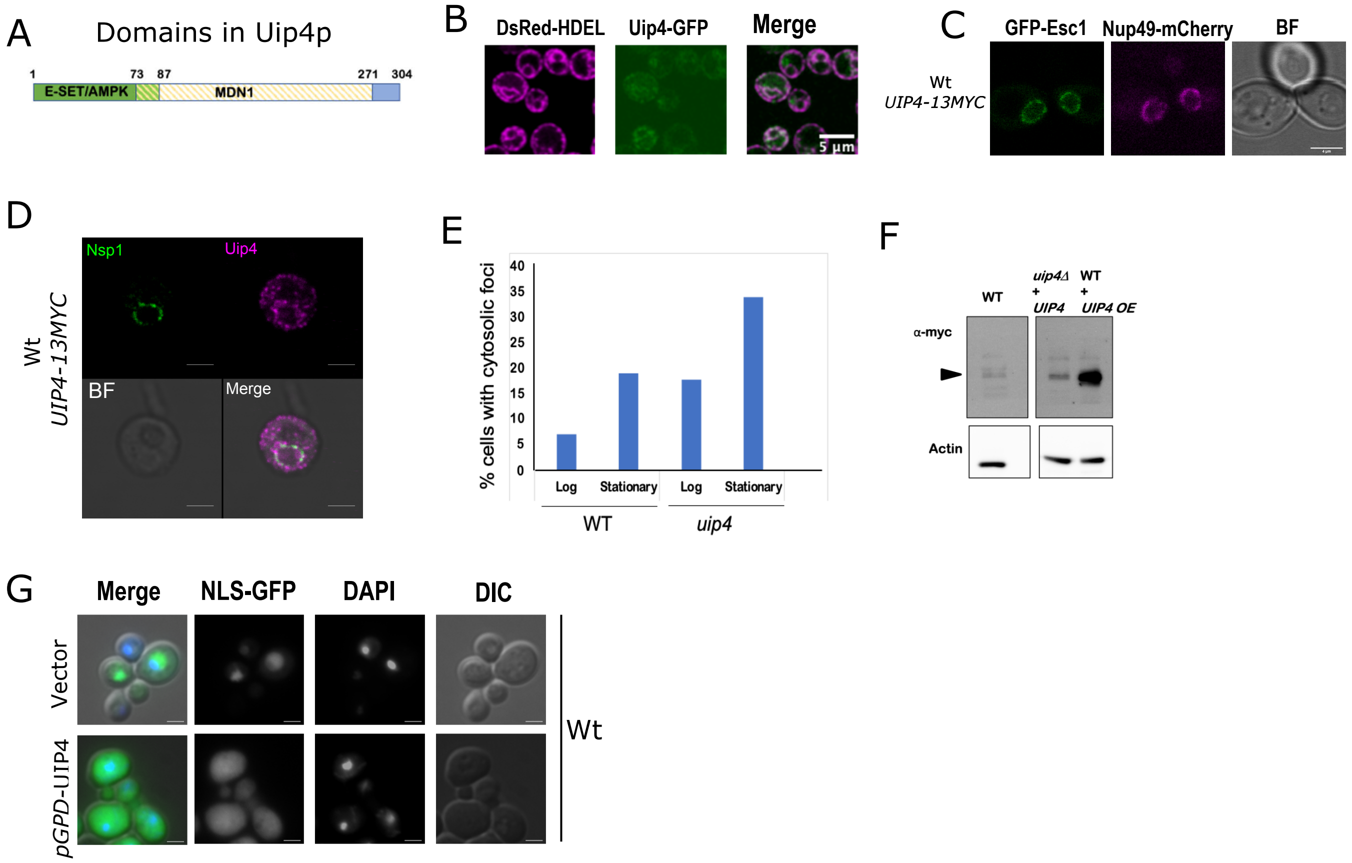

FigureS4.

A. Domain map of Uip4 showing position of E-set AMPK and the MDN1 domain.

B. Uip4 was tagged with a GFP at its C-terminal in strain co-expressing DsRedHDEL from *TRP1* locus. The micrograph shows Uip4 localization (green), merged with a known ER localized signal (red). Scale 5µm

C. Strain expressing Uip4 tagged with C-terminal 13xMyc epitope at endogenous loci was co-transformed with plasmids expressing GFP-Esc1 and Nup49-mCherry. Live cells were imaged and representative images are shown. Scale-2µm

D. Indirect immunofluorescence using α-myc was performed in the strain carrying UIP4-13MYC to check the localization of Uip4. Co-staining with a NE marker Nsp1 is shown. Scale 2µm

E. The bar graph represents the fraction of cells in the indicated strain harvested from either mid-log or stationary phase showing cytosolic spots of GFP-Nup49. ~100 cells from 2 independent experiments were counted.

F. Western blot analysis was done to confirm Uip4 overexpression (OE) from *pGPD* as compared to the expression from endogenous promoter. α-myc was used to detect Uip4. Actin is the loading control.

G. Nuclear import was tested in Wt cells bearing NLS-2X GFP plasmid, co-transformed with either empty vector or vector expressing UIP4 from *pGPD*. DAPI staining was used to define the nucleus. Scale-2µm
